# Adjuvant therapeutic vaccination in patients with non-small cell lung cancer made lymphopenic and reconstituted with autologous PBMC: first clinical experience and evidence of an immune response

**DOI:** 10.1186/1479-5876-5-43

**Published:** 2007-09-14

**Authors:** Dominik Rüttinger, Natasja K van den Engel, Hauke Winter, Marcus Schlemmer, Heike Pohla, Stefanie Grützner, Beate Wagner, Dolores J Schendel, Bernard A Fox, K-W Jauch, Rudolf A Hatz

**Affiliations:** 1 Department of Surgery, Grosshadern Medical Center, Ludwig-Maximilians-University, Marchioninistrasse 15, 81377 Munich, Germany; 2 Department of Internal Medicine III, Grosshadern Medical Center, Ludwig-Maximilians-University, Munich, Germany; 3 Laboratory of Tumor Immunology, Ludwig-Maximilians-University, Munich, Germany; 4 Institute of Molecular Immunology, and Clinical Cooperation Group "Immune Monitoring", GSF National Research Center for Environment and Health, Munich, Germany; 5 Department of Transfusion Medicine, Grosshadern Medical Center, Ludwig-Maximilians-University, Munich, Germany; 6 Robert W. Franz Cancer Research Center, Earle A. Chiles Research Institute, Providence Portland Medical Center, Portland, Oregon, USA

## Abstract

**Background:**

Given the considerable toxicity and modest benefit of adjuvant chemotherapy for non-small cell lung cancer (NSCLC), there is clearly a need for new treatment modalities in the adjuvant setting. Active specific immunotherapy may represent such an option. However, clinical responses have been rare so far. Manipulating the host by inducing lymphopenia before vaccination resulted in a magnification of the immune response in the preclinical setting. To evaluate feasibility and safety of an irradiated, autologous tumor cell vaccine given following induction of lymphopenia by chemotherapy and reinfusion of autologous peripheral blood mononuclear cells (PBMC), we are currently conducting a pilot-phase I clinical trial in patients with NSCLC following surgical resection. This paper reports on the first clinical experience and evidence of an immune response in patients suffering from NSCLC.

**Methods:**

NSCLC patients stages I-IIIA are recruited. Vaccines are generated from their resected lung specimens. Patients undergo leukapheresis to harvest their PBMC prior to or following the surgical procedure. Furthermore, patients receive preparative chemotherapy (cyclophosphamide 350 mg/m^2 ^and fludarabine 20 mg/m^2 ^on 3 consecutive days) for induction of lymphopenia followed by reconstitution with their autologous PBMC. Vaccines are administered intradermally on day 1 following reconstitution and every two weeks for a total of up to five vaccinations. Granulocyte-macrophage-colony-stimulating-factor (GM-CSF) is given continuously (at a rate of 50 μg/24 h) at the site of vaccination via minipump for six consecutive days after each vaccination.

**Results:**

To date, vaccines were successfully manufactured for 4 of 4 patients. The most common toxicities were local injection-site reactions and mild constitutional symptoms. Immune responses to chemotherapy, reconstitution and vaccination are measured by vaccine site and delayed type hypersensitivity (DTH) skin reactions. One patient developed positive DTH skin tests so far. Immunohistochemical assessment of punch biopsies taken at the local vaccine site reaction revealed a dense lymphocyte infiltrate. Further immunohistochemical differentiation showed that CD1a+ cells had been attracted to the vaccine site as well as predominantly CD4+ lymphocytes. The 3-day combination chemotherapy consisting of cyclophosphamide and fludarabine induced a profound lymphopenia in all patients. Sequential FACS analysis revealed that different T cell subsets (CD4, CD8, CD4CD25) as well as granulocytes, B cells and NK cells were significantly reduced. Here, we report on clinical safety and feasibility of this vaccination approach during lymphoid recovery and demonstrate a patient example.

**Conclusion:**

Thus far, all vaccines were well tolerated. The overall trial design seems safe and feasible. Vaccine site reactions associated with infusion of GM-CSF via mini-pump are consistent with the postulated mechanism of action. More detailed immune-monitoring is required to evaluate a potential systemic immune response. Further studies to exploit homeostasis-driven T cell proliferation for the induction of a specific anti-tumor immune response in this clinical setting are warranted.

## Background

There are approximately 170,000 new cases of lung cancer in the United States each year and almost 160,000 annual deaths, clearly demonstrating that despite progress in the treatment of this disease over the past two decades, there are still few long-term survivors: only 12% of all patients will ever be cured of this devastating disease. Clearly, there is still an unmet medical need for new adjuvant therapies that demonstrate efficacy in lung cancer with less associated toxicity than chemotherapy. The unfortunate reality is, however, that the field of lung cancer immunotherapy, which may provide a valid alternative for cytotoxic agents, lags behind similar efforts with other kinds of tumors, primarily melanoma, prostate and breast carcinoma.

The sources of tumor antigens in cancer vaccine strategies include isolated peptides, tumor cell lysates, or whole tumor cells from either autologous tumor or established allogeneic tumor cell lines. In the case of NSCLC, the immunodominant antigens are unknown, making development of relevant peptide vaccines challenging.

Furthermore, NSCLC is composed of a mixture of histological subtypes, making selection of one or two relevant allogeneic cell lines that will encompass the full antigenic diversity of NSCLC difficult.

The rationale for cancer vaccine therapy is to modify relatively non-immunogenic tumors to induce a tumor-specific immune response in the host. Cancer cell vaccines, genetically modified to secrete immunomodulatory cytokines, have generated potent anti-tumor immunity in pre-clinical animal models of melanoma, lymphoma, colon, renal, lung, and prostate cancer. A comparison involving multiple cytokine genes found that granulocyte-macrophage colony-stimulating factor (GM-CSF) gene-transduced vaccines were by far the most potent inducers of long-lasting specific tumor immunity in one animal model [1]. Secretion of GM-CSF by genetically-modified tumor cells stimulates cytokine release at the vaccine site to activate antigen-presenting cells, which prime CD4+ and CD8+ T cells to recognize circulating tumor-associated antigens, thereby inducing a tumor-specific cellular immune response. Induction of a tumor-specific humeral immune response and activation of a granulocytic (e.g., eosinophils and neutrophils) inflammatory reaction may also contribute to the efficacy of this approach. Early-phase human clinical trials of GM-CSF-secreting autologous tumor cells in lung cancer have demonstrated the relative safety of this treatment approach as well as the induction of tumor-specific immune responses and evidence of anti-tumor activity [2,3]. However, objective response rates are still low.

It has previously been reported that immune modulating doses of cyclophosphamide enhance vaccine-induced anti-tumor immune responses by inhibiting suppressor T cell activity [4,5]. Several clinical trials have since combined cyclophosphamide with vaccination. Cyclophosphamide and another chemotherapeutic agent, fludarabine, have been administered together in the treatment of CLL [6] and low-grade non-Hodgkin's Lymphoma [7]. The same combination has been administered in a clinical study of non-myeloablative chemotherapy and adoptive transfer of highly selected tumor-reactive T cells to treat patients with metastatic melanoma [8]. These patients were reinfused with in vitro expanded tumor-infiltrating T cells followed by IL-2 therapy. This study clearly documented the safety of this conditioning regimen in combination with adoptive T cell transfer and demonstrated an objective response in 6 of 13 patients.

The adoptive transfer of in vitro expanded tumor-infiltrating lymphocytes exceeds, however, the technical and financial resources of most laboratories. We hypothesized, therefore, that inhibiting the activity of regulatory T cells and exploiting the increased sensitivity of lymphocytes to respond to antigenic stimuli when placed under conditions of homeostasis-driven proliferation [9-12] might be possible when preparative chemotherapy, reconstitution with autologous PBMC and vaccination with irradiated, autologous tumor cells are combined. In preclinical experiments, this was modeled by vaccinating lymphopenic mice with a GM-CSF gene-modified melanoma cell line following reconstitution with naïve spleen cells. Subsequent examination of tumor vaccine-draining lymph node T cells revealed a substantial increase in the frequency of activated T cells [13]. Following in vitro activation these T cells contained a significantly elevated frequency of tumor-specific CD4+ and CD8+ T cells with augmented function in vitro and therapeutic efficacy in vivo. Three possible explanations for the beneficial effect of combining lymphopenia with reconstitution and vaccination are: 1) creation of space (physical space as well as reduced competition for cytokines), 2) depletion of regulatory T cells and 3) direct anti-tumor effect ("softening-up the tumor").

Based upon these observations we initiated a pilot-Phase I clinical trial for patients with NSCLC made lymphopenic by preparative chemotherapy and reconstituted with autologous PBMC prior to vaccination with their autologous tumor cells. Here, we present our first clinical experience and evidence of an immune response.

## Methods

### Patients

This pilot-phase I clinical trial is performed at the Department of Surgery – Grosshadern Medical Center of the Ludwig-Maximilians-University (LMU) Munich, Germany. The clinical trial protocol received approval of the ethics committee of the LMU Munich (registration number 291/04) and the Bavarian government. All patients enrolled sign informed consent and all procedures are carried out in compliance with the Helsinki declaration. Patients are eligible for enrollment if they have histologically confirmed NSCLC stages I-IIIA (surgically resectable), an Eastern Cooperative Oncology Group performance status of 0 or 1, age ≥ 18 years, an oxygen saturation ≥ 90% on ≤ 2L supplemental oxygen, a forced expiratory volume in one second (FEV1) ≥ 40% of predicted, a CD4+ cell count > 200/mm^3^, and are ≥ 4 weeks from chemotherapy and ≥ 2 weeks from radiotherapy and systemic corticosteroid treatment. Patients are excluded if they are pregnant, have any known hypersensitivity to components of the vaccine or any of the study drugs or have any uncontrolled medical problems which are considered high risk to take part in this clinical trial. Patients are further excluded for the following reasons: previous treatment with cancer vaccines or gene therapy, active autoimmune disease, or infection with human immunodeficiency virus. All patients undergo staging scans, pulmonary function testing and routine hematology and chemistry analysis as considered standard prior to open lung surgery. Additional hematological criteria have to be met prior to collection of the leukaphereses for immune-monitoring and reconstitution of the patients: Total white blood cell count (WBC) ≥ 2500/mm^3 ^and/or absolute neutrophil count (ANC) > 1000/mm^3^, hemoglobin > 8.0 g/dL, platelet count > 100,000/mm^3^, and Hct > 24%.

### Vaccine preparation

Tumor procurement is performed according to accepted standards of surgical practice. This protocol requires complete tumor resection. A tissue sample is submitted for definite histopathological assessment and confirmation of the diagnosis of NSCLC. The remaining tumor is processed immediately to a single-cell suspension by physical mincing and enzymatic digestion using a triple enzyme solution containing 0.02 mg/ml deoxyribonuclease I, 0.25 mg/ml collagenase and 0.1 mg/ml hyaluronidase (Sigma-Aldrich, Taufkirchen, Germany). Cells are washed and irradiated at 10,000 rads to prevent cell proliferation. Cells are then aliquoted into vials in a formulation containing 10% dimethylsulfoxide (DMSO) (Sigma-Aldrich, Taufkirchen, Germany), 45% RPMI-1640 medium (Cambrex Bio Science, Verviers, Belgium) and 45% autologous serum of the corresponding patient, and are then cryopreserved. Vaccine cells are tested for tumor cell dose, viability, endotoxin, and sterility. Individual patient vaccines that do not meet release criteria are not be used and the corresponding patients are not enrolled for vaccine treatment. Before clinical administration, cryopreserved cells are thawed, washed extensively, and resuspended in 1 mL of sterile saline for the vaccines and 0.5 mL for cells used in delayed-type hypersensitivity testing. Additional tumor cells (depending on the total number of tumor cells available) are stored frozen or placed into culture for immune-monitoring purposes. The vaccine dose is individualized for each patient based upon tumor cell yield but is required to be at least 5 × 10^6 ^tumor cells/dose. The dose for each vaccination is equivalent. The minimum total vaccine dose required for treatment is 25 × 10^6 ^tumor cells (5 × 10^6 ^cells × 5 vaccinations). Aliquots of 1 × 10^6 ^tumor cells are set aside for use in DTH testing.

### Treatment and evaluation

**Figure 1:**
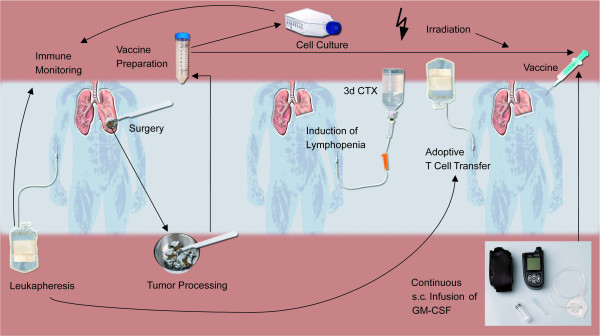
**Outline of the clinical trial protocol**. Non-small cell lung cancer patients stages I-IIIa undergo curative surgery to prepare an autologous vaccine. The autologous tumor cells are physically minced and digested by a triple enzyme solution. The vaccine is then irradiated at 10,000 rads, tested for sterility and cryopreserved. Immediately following the intradermal vaccination (biweekly, total of up to five vaccinations) GM-CSF is infused subcutaneously for 6 days at a rate of 50 μg/24 h. Prior to vaccination plus GM-CSF administration, lymphopenia is induced by a 3-day combination chemotherapy (cyclophosphamide 350 mg/m^2 ^and fludarabine 20 mg/m^2^) followed by reconstitution with autologous peripheral blood mononuclear cells. Two additional leukaphereses are harvested pre- and post-vaccination for immunemonitoring purposes. (CTX: chemotherapy)

**Figure 2:**
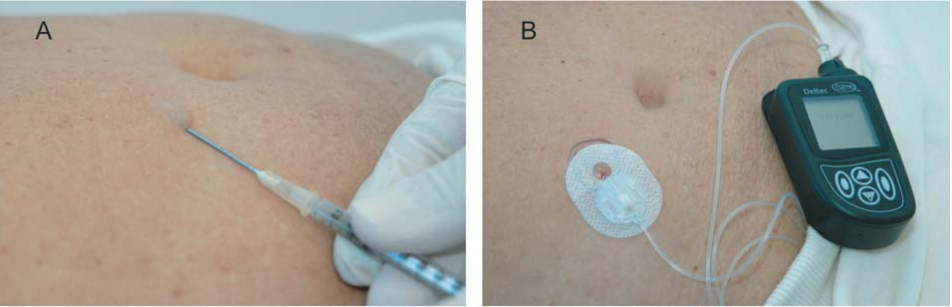
**A. Intradermal application on the abdominal wall; B. Continuous infusion of GM-CSF at the vaccine site using a minipump**. Vaccines are administered intradermally on alternating sides of the abdominal wall (A). Before clinical administration, the irradiated autologous tumor cells are thawed, washed extensively, and resuspended in 1 mL of sterile saline. Immediately following the vaccine application a catheter is placed at the vaccine site and GM-CSF is infused continuously at a rate of 50 μg/24 h for a total of 6 days using an osmotic minipump (Smiths Medical, Kirchseeon, Germany) (B).

The treatment schedule is illustrated in Figure 1. Based on the available preclinical and clinical data, we have initiated this pilot study assessing the safety and efficacy of an autologous NSCLC tumor cell vaccine administered with immunomodulatory doses of cyclophosphamide and fludarabine. In this protocol the vaccine is administered intradermally (Figure 2). The intradermal route of administration was selected in order to ensure close approximation of the antigen and GM-CSF to the intradermal dendritic cells which are involved in tumor-associated antigen presentation to host T cells.

Following the enrollment screening, all patients signed informed consent and underwent the standard surgical procedure which consisted of (bi-) lobectomy and mediastinal/hilar lymphadenectomy. Following sufficient recovery from the surgical procedure two leukaphereses were harvested, one for later reconstitution and one for immune monitoring purposes. Leukaphereses were performed using a continuous flow cell separator (Cobe Spectra, Gambro GmbH, Planegg, Germany). Aphareses products were analyzed by flow cytometry (see methods below), frozen in DMSO and stored above liquid nitrogen according good manufacturing practice (GMP) until reinfusion. All patients then received preparative chemotherapy consisting of cyclophosphamide (350 mg/m^2 ^IV) and fludarabine (20 mg/m^2 ^IV) at days 1–3 prior to the first vaccination. The induction of lymphopenia was followed by reinfusion of PBMC (leukapheresis product) and the intradermal administration of the first vaccine. Immediately following the vaccine application, the subcutaneous catheter for instillation of GM-CSF (Leukine^®^, Berlex Laboratories Inc., Richmond, CA, USA) via the infusion minipump is placed locally in the center area of the vaccine site (Fig. 2). GM-CSF is infused over six full days at a rate of 50 μg/24 h.

Patients receive intradermal vaccine injections every two weeks for a total of up to five vaccine cycles. The dose is individualized for each patient based on vaccine yield and ranges from 14 × 10^6 ^– 100 × 10^6 ^tumor cells/vaccination. Immunologic response to vaccination is monitored by delayed-type hypersensitivity (DTH) skin testing to injections of autologous tumor cells at base line and 48 h following the fourth vaccine. After completion of the trial protocol a third leukapheresis is harvested for immunemonitoring purposes. Patients then continue to have monthly immune surveillance in addition to their standard clinical observation every 3 months.

### Delayed type hypersensitivity (DTH) tests

To evaluate cell-mediated reactivity induced by application of autologous tumor cells DTH tests were perfomed at baseline (before induction of lymphopenia) and 48 h following the fourth vaccine. DTH skin tests consisted of 0.5 mL containing an estimate of 10^6 ^cells. The cells were injected intradermally into the upper arm and read 48 h later. DTH skin tests were considered positive if an erythematous and indurated area measuring at least 5 mm appeared.

### Immunohistochemistry and flow cytometry

Punch biopsies were taken at the vaccine site and an unaffected skin site as a control. In brief, tissues were fixed in 10% neutral buffered formalin, processed routinely and embedded in paraffin. Specimens were stained with hematoxylin/eosin and assessed immunohistochemically for CD3, CD4, CD8 and CD1a (as a marker for dendritic cells) using the corresponding monoclonal antibodies (Biozol GmbH, Eching, Germany).

Based on the available preclinical and early clinical data, we hypothesized that vaccination during a phase of lymphoid recovery may result in a greater tumor-specific immune response. To investigate whether our conditioning regimen with preparative chemotherapy and reinfusion of autologous PBMC actually induced a homeostatic-driven lymphocyte proliferation phase, we assessed different lymphocyte subsets over the time course of the clinical trial protocol using fluorescence activated cell sorting (FACS). PBMC were stained for 30 min, washed in FACS buffer, and fixed in 1% paraformaldehyde in PBS. Monoclonal antibodies and isotype controls were used for CD3, CD4, CD8, CD16, CD19, CD25, CD56 (BD Biosciences, Heidelberg, Germany) Acquisition of data was performed on FACS-Calibur flow cytometer (BD Biosciences, Heidelberg, Germany), and data was analyzed using WINMDI28 software [14].

## Results

### Patient characteristics

To date, 4 patients were enrolled in the trial, 2 males and 2 females. The age range at diagnosis was 49 – 78. All patients had a performance status of 0 according to the Eastern Cooperative Oncology Group (ECOG) scale. None of the patients showed evidence of metastatic disease as evaluated by CTscan, PETscan and/or skeletal szintigraphy. Three patients underwent lobectomy and 1 patient a bilobectomy with all patients getting a standard systematic hilar/mediastinal lymphadenectomy. Different histopathologic subgroups of NSCLC were represented with adenocarcinoma, squamous cell carcinoma and large-cell carcinoma. There were no serious adverse events associated with the surgical procedures. None of the patients had prior systemic therapy including chemotherapy and immunotherapy. One patient withdrew by choice before initiation of the treatment phase. Thus far, 3 patients completed the study protocol including preparative chemotherapy, reconstitution of autologous PBMC, vaccination and post-vaccination leukapheresis for immunemonitoring purposes. All patients on the trial refused standard adjuvant chemotherapy as recommended by the medical oncologist. The longest follow up period with 17 months was that of the first patient enrolled. Table 1 summarizes the baseline patient characteristics.

**Table 1:** Patient characteristics

**Patient**	**Gender/Age**	**Prior Therapies**	**Surgery^*a*^**	**Histology**	**Tumor stage**	**Performance status^*c*^**
1	F/78	none	upper bilobectomy	adenocarcinoma	IIIA	0
2	F/47	none	lobectomy	squamous-cell carcinoma	IB	0
3	M/65	none	lobectomy	large-cell carcinoma	IIA	0
4 ^*b*^	M/77	none	lobectomy	adenocarcinoma	IB	0

^*a *^all patients received standard systematic hilar/mediastinal lymphadenectomy

^*b *^patient withdrew by choice before the treatment phase

^*c *^Eastern Cooperative Oncology Group (ECOG) scale

### Vaccine production, administration and delivery of GM-CSF

Solid tumors were processed to a single cell suspension by mechanical mincing and enzymatic digestion. Vaccine production was successful in 4 of 4 patients. All tumor specimens were processed fresh. Wet tumor weight ranged from 11 – 31 grams (mean: 20 grams). The average tumor cell number obtained was 27.9 × 10^8 ^cells per gram wet tumor (range 1.8 – 105 × 10^8^). The viability of cells as assessed by trypan blue staining ranged from 1% to 55%. Sterility of the vaccine following irradiation at 10,000 rads was confirmed in all cases. The average number of days from tumor harvest to the first vaccination was 52.3 (range 48 – 57). All tumor harvests met the minimum requirement for tumor cell number of 5 × 10^6 ^per vaccine (total of 25 × 10^6 ^for all 5 vaccinations). Table 2 summarizes the vaccination details. Following each vaccination GM-CSF (Leukine^®^, Berlex Laboratories Inc., Richmond, CA, USA) was infused subcutaneously at the vaccination site for 6 full days at a rate 50 μg/24 h using an osmotic minipump (Smiths Medical, Kirchseeon, Germany) (Figure 2). This technique turned out to be technically feasible and easily tolerable for the patients.

**Table 2:** Vaccination details and characteristics of reinfused mononuclear cells

**Reinfused leukapheresis product^*a*^**							
**Patient**	**Tumor weight [gram]**	**Tumor cell yield**	**No. of tumor cells/vaccine**	**No. of nuclear cells**	**% lympocytes**	**% CD3^+*c*^**	**%CD4^+^/%CD8^+*c*^**

1	11	1.8 × 10^8^	2 × 10^7^	0.9 × 10^10^	39	81	65/19
2	31	105 × 10^8^	10 × 10^7^	2.1 × 10^10^	53	79	57/22
3	24	1.9 × 10^8^	1.4 × 10^7^	0.9 × 10^10^	71	65	52/12
4 ^*b*^	14	2.8 × 10^8^	n.a.	n.a.	n.a.	n.a.	n.a.

^*a *^PBMC were reinfused following 3 days of preparative chemotherapy (cyclophosphamide 350 mg/m^2 ^and fludarabine 20 mg/m^2^)

^*b *^patient withdrew by choice before the treatment phase

^*c *^shown as percentage of lymphocytes

n.a. not applicable

### Reconstitution of lymphopenic patients and lymphoid recovery

Following 3 days of conditioning chemotherapy with cyclophosphamide (350 mg/m^2^) and fludarabine (20 mg/m^2^) all patients received the leukapheresis product harvested earlier. The first vaccine was administered 24 h following the reconstitution with the reinfusion of PBMC being administered 48 h following the last dose of chemotherapy. A mean of 1.3 × 10^10 ^nuclear cells (range 0.9 – 2.1 × 10^10 ^cells) was reinfused. An average of 54% of the cells (range 39 – 71%) were recognized as lymphocytes using FACS analysis. 75% of the lymphocytes were CD3 positive (range 65 – 81%) with 58% (range 52 – 65%) and 18% (range 12 – 22%) being CD4 and CD8 positive, respectively. Table 2 summarizes the characteristics of the reinfused mononuclear cells.

FACS analysis of PBMC was performed at different time points throughout the course of the treatment phase to investigate whether the chosen protocol actually induces a homeostatic-driven lymphocyte proliferation/recovery phase that may be taken advantage of by applicating repeated tumor cell vaccinations. The combination chemotherapy induced a profound lymphopenia in all patients with low total white blood cell counts and absolute lymphocyte counts. All of the determined CD3 positive cell subsets (CD4, CD8, CD4CD25) were affected. Neutrophil counts recovered to pre-chemotherapy levels within 30 days. Recovery of different T cell subsets (CD4, CD8, CD4CD25) was slower in all patients but varied inter-individually. The post-chemotherapy increase in CD4 numbers followed the same kinetics as the other subsets. One patient experience an extended depression of CD19-positive B lymphocytes, whereas NK cells (CD3-CD16+CD56+) came back within 30 days in all patients. Red blood cell and platelet counts were not affected significantly. A patient example is given below.

### Tumor-related immunity

**Figure 3:**
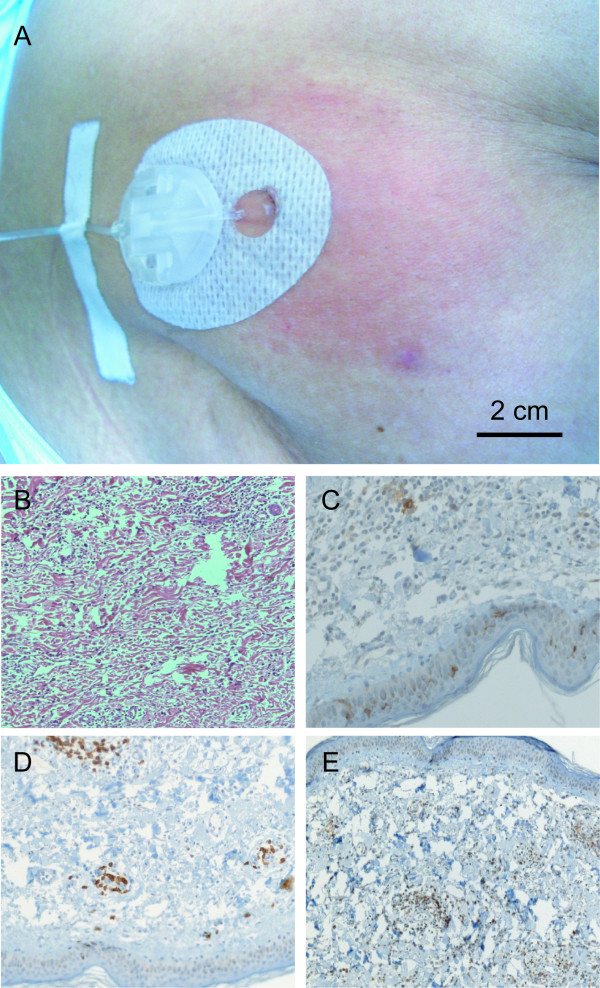
**Local vaccine site reaction and immunohistochemistry of punch biopsies**. (A) Typical vaccine site reaction 48 h following the third vaccination. Clinically, the reaction was characterized by erythema, induration and pruritus. Note the catheter for local infusion of GM-CSF placed in the center of the vaccine application site. Punch biopsies were taken at the reaction site and assessed immunohistochemically. Histopathological assessment of punch biopsy specimens taken at the local vaccine site reaction 48 h following the third vaccine revealed a dense infiltrate consisting predominantly of macrophages, eosinophils, neutrophils and lymphocytes (B, ×100). In contrast to unrelated skin sites, CD1a-positive dendritic cells were present at the vaccine site (C, ×400). Further immunohistochemical staining showed that most of the CD3-positive lymphocytes (D, ×400) in the patient with the strongest vaccine site reaction were CD4-positive cells (E, ×100).

Injections of irradiated, autologous NSCLC cells combined with local infusion of GM-CSF following preparative chemotherapy and reconstitution with autologous PBMC elicited local vaccine site reactions in all patients. Intensity and frequency of the responses varied but generally increased with the number of vaccines administered. Clinically, the reactions were characterized by local erythema and induration (Figure 3). One patient with a strong local vaccine site reaction also reported recall responses at the site of a previous injection and the baseline DTH injection site.

Irradiated, dissociated, autologous NSCLC cells were available for DTH testing in all patients. Although 3/3 patients developed local vaccine site reactions, a positive DTH skin test was observed in only one patient 48 h following the fourth vaccine. None of the DTH tests were positive before the vaccination phase.

Histopathological assessment of punch biopsy specimens taken at the local vaccine site reaction 48 h following the third vaccine revealed a dense infiltrate consisting predominantly of macrophages, eosinophils, neutrophils and lymphocytes (Figure 3). In contrast to unrelated skin sites, CD1a-positive dendritic cells were present at the vaccine site. Further immunohistochemical staining showed that most of the infiltrating lymphocytes in the patient with the strongest vaccine site reaction were CD4-positive cells.

### Toxicity

Vaccination consistently induced grade 1 to 2 erythema and induration at the injection sites on the abdominal wall. Local pruritus was also reported. Occasional grade 1 to 2 fatigue and flu-like symptoms were observed. There were no significant hepatic, renal, pulmonary, cardiac, hematologic, gastrointestinal or neurologic toxicities attributable either to preparative chemotherapy, reinfusion of autologous PBMC or the vaccine. No severe autoimmune phenomena such as pericarditis, hypophysitis or vasculitis were noted. However, one patient reported persistent myalgias and joint pain. In this patient, a transient elevation of markers such as rheumatoid factor and antinuclear antibodies was detectable, potentially indicating a temporary autoimmune activation.

Hematologic toxicities due to the conditioning chemotherapy were transient and did not require transfusion therapy. Low absolute neutrophil counts, low absolute lymphocyte counts, and the transient depression of CD19-positive B cells were observed in all patients. No typical opportunistic infections such as Herpes zoster or Pneumocystis carinii were detected.

No serious adverse events attributable to the local infusion of GM-CSF were detected.

### Clinical course and patient example

In the present study, NSCLC patients were vaccinated as an adjuvant therapy without detectable tumor manifestation following surgery. Therefore, no target lesions were available for monitoring of a potential clinical response to treatment. Nevertheless, all patients on this trial were assessed at 3-months intervals by means of clinical examination, determination of specific tumor markers and radiographs (CTscan). To date, all patients on the trial remain without relapse with the longest follow-up period being 17 months for the first enrolled patient (patient 1, Table 1).

**Figure 4:**
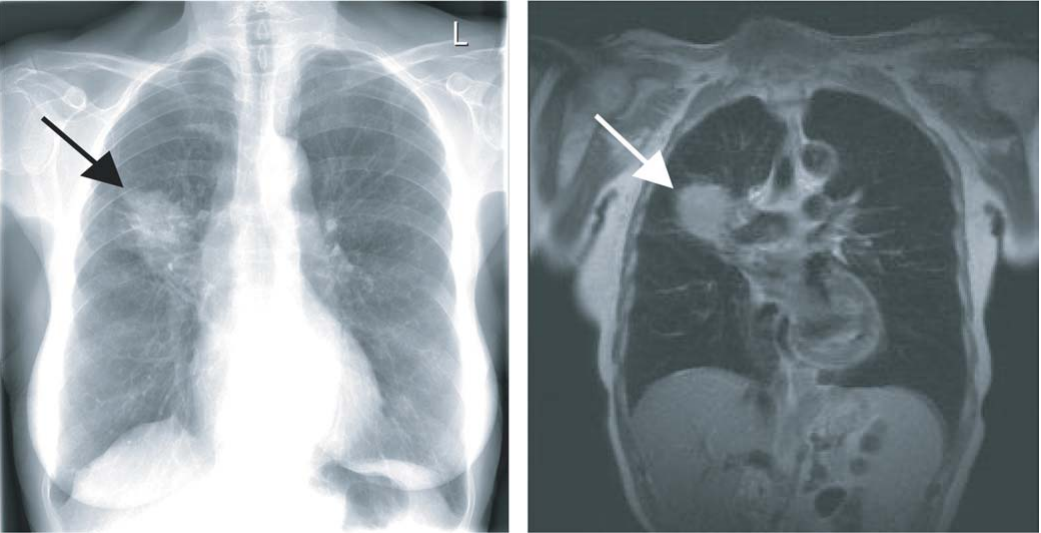
**Baseline chest x-ray (left) and MRI scan (right) of patient 1**. Note the large tumor originating from the upper right lobe of the lung (arrows). The patient was resected with an upper bilobectomy (pT2 pN2, IIIA) and systematic lymphadenectomy and the diagnosis of NSCLC (adenocarcinoma) was confirmed.

Figure 4 shows the chest X-ray and thoracic MRI of patient 1 with an obvious tumor mass originating from the upper lobe of the right lung. The patient was resected with an upper bilobectomy (pT2 pN2, IIIA) and refused the standard adjuvant treatment for resected stage IIIA NSCLC consisting of chemotherapy. Following successful vaccine production and recovery from the surgical procedure the patient underwent preparative chemotherapy, reconstitution with autologous PBMC and repeated vaccinations as described above.

**Figure 5:**
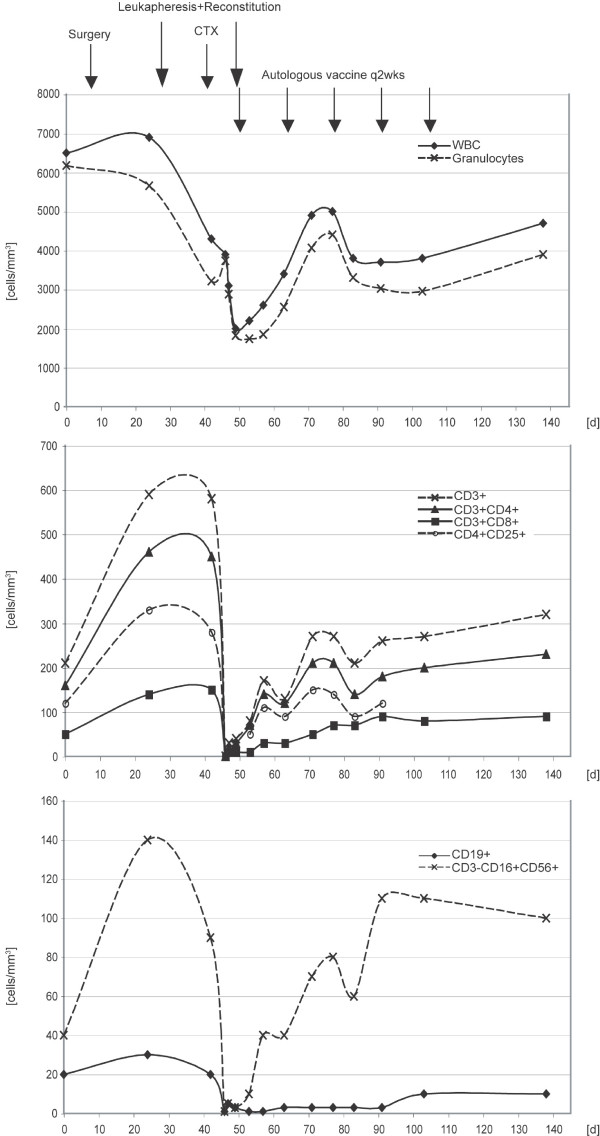
**Analysis of absolute white blood cell count (WBC), neutrophil count and flow-cytometric assessment of lymphocytes, T cell subsets and natural killer cells in patient 1 during the treatment phase**. After preparative chemotherapy (CTX) (cyclophosphamide 350 mg/m^2 ^and fludarabine 20 mg/m^2^) the patient was reconstituted with 0.9 × 10^10 ^autologous PBMC (5.05 × 10^7 ^CD3-positive cells/kg) followed by 5 cycles of the autologous tumor cell vaccine. All of the determined CD3-positive cell subsets (CD4, CD8, CD4CD25) were affected by cyclophosphomide and fludarabine. As seen in all patients, neutrophil counts recovered to pre-chemotherapy levels within 30 days. Recovery of different T cell subsets (CD4, CD8, CD4CD25) was slower than normalization of neutrophil counts in all patients but varied inter-individually. The post-chemotherapy increase in CD4 numbers followed the same kinetics as the other subsets with no extended depression as one might expect following fludarabine treatment. Patient 1 experienced an extended depression of CD19-positive B lymphocytes, whereas NK cells (CD3-CD16+CD56+) recovered within 30 days (as observed in all patients). Additional leukaphereses were harvested prior to preparative chemotherapy and after the vaccination phase.

Figure 5 illustrates the time course of lymphoid recovery following induction of lymphopenia by preparative chemotherapy as seen in patient 1. The patient was reconstituted with 0.9 × 10^10 ^autologous PBMC (5.05 × 10^7 ^CD3-positive cells/kg) followed by 5 cycles of autologous tumor cell vaccine. All of the determined CD3-positive cell subsets (CD4, CD8, CD4CD25) were affected by cyclophosphamide and fludarabine. As seen in all patients, neutrophil counts recovered to pre-chemotherapy levels within 30 days. Recovery of different T cell subsets (CD4, CD8, CD4CD25) was slower than normalization of neutrophil counts in all patients but varied inter-individually. The post-chemotherapy increase in CD4 numbers followed the same kinetics as the other subsets with no extended depression as one might expect following fludarabine treatment. Patient 1 experienced an extended depression of CD19-positive B lymphocytes, whereas NK cells (CD3-CD16+CD56+) returned within 30 days (as observed in all patients).

## Discussion

Lung cancer vaccines have progressed from pre-clinical to clinical studies [3,15,16] and potentially represent a novel and safe form of treatment that is urgently needed considering the toxicity and modest benefit of existing adjuvant therapies for NSCLC. However, effectiveness of lung cancer vaccines has yet to be established. We hypothesized that vaccines for lung cancer (and other cancers) fail because the magnitude of the anti-tumor response is insufficient to mediate tumor regression. Elimination of regulatory T cells (Tregs) and "creation of space" prior to vaccination may augment the immune response and vaccine efficacy. Here, we report for the first time on early clinical results of an autologous lung cancer vaccine administered to patients who have been made lymphopenic by preparative chemotherapy and reconstituted with autologous PBMC. To our knowledge, this is also the first attempt to use an osmotic minipump for the continuous delivery of GM-CSF as an adjuvant to the vaccine site.

### Adjuvant vaccination

With few exceptions, most early phase clinical trials evaluating lung cancer vaccines enroll patients with late-stage disease (stages IIIA-IV). In these patients, a median survival of 11 months may be achieved using established treatment modalities such as chemotherapy and radiotherapy. Tumor-induced immune suppression and compromised performance status may contribute to the limited efficacy of active-specific immunotherapy for this group of patients. It seems obvious to move to the adjuvant setting where immunosuppressive influences may have been removed through tumor resection and potentially remaining tumor cells are referred to as minimal residual disease. In accordance with this hypothesis, patient 1 on our trial failed the first enrollment screening with less than 200 CD4+ cells/mm^3^, but was eligible following surgery when her CD4 cell count had recovered, indicating a possible immunosuppressive effect of the tumor (Figure 5). Other groups also investigated adjuvant vaccination for lung cancer such as recombinant MAGE-A3 fusion protein vaccination where a large Phase III clinical trial is currently being initiated [17].

### Vaccinating during lymphoid recovery

Recently, different groups have identified an approach that exploits the increased sensitivity of lymphocytes to respond to antigenic stimuli when they are placed under conditions of homeostasis-driven proliferation [9-13,18] (reviewed in [19,20]). In the preclinical setting, this was modeled by vaccinating lymphopenic mice with a GM-CSF gene-modified melanoma cell line following reconstitution with naïve spleen cells. The reconstitution step provided a broad repertoire of non-damaged T cells to the vaccinated host. Subsequent examination of tumor vaccine-draining lymph node T cells from reconstituted-lymphopenic mice revealed a substantial increase in the frequency of activated T cells when compared to lymph node T cells from normal mice [13]. Following in vitro activation these T cells contained a significantly elevated frequency of tumor-specific CD4 and CD8 T cells with augmented function in vitro and therapeutic efficacy in vivo. Three explanations for the beneficial effect of combining lymphopenia with reconstitution and vaccination may be: 1) creation of space (physical space as well as reduced competition for cytokines such as IL-7 or IL-15 [12,21]), 2) depletion of regulatory T cells [22] and 3) direct anti-tumor effect (softening-up the tumor). The combination chemotherapy used in our trial (cyclophosphamide/fludarabine) has little anti-NSCLC activity and a direct anti-tumor effect is unlikely in this adjuvant setting. Nonetheless we employed this strategy because of its immune amplifying potential. Dudley and coworkers [8] pretreated melanoma patients with a "non-myeloablative" chemotherapy regimen consisting of 2 days of cyclophosphamide at 60 mg per kg of body weight, followed by 5 days of fludarabine at 25 mg/m^2^. On the day after the final dose of fludarabine, when circulating lymphocyte and neutrophil counts had dropped to less than 20/mm^3^, each patient received an intravenous infusion of autologous tumor-infiltrating lymphocytes (TIL) that had been expanded in vitro. Infused cells expanded and persisted in a majority of patients and were associated with a higher objective response rate than adoptive immunotherapy with TIL alone. A single, low, intravenous dose (usually 300 mg/m^2 ^to a maximum of 600 mg) of cyclophosphamide was administered before immunotherapy in other studies [11,15,23]. This enhanced the effect of immunotherapy. In various animal models, cyclophosphamide has demonstrated its ability to augment DTH responses, increase antibody production, abrogate tolerance, and potentiate antitumor immunity [11,23]. With 3 consecutive days of 350 mg/m^2^cyclophosphamide and 20 mg/m^2 ^fludarabine, we chose a dose lower than that of Dudley et al. but more potent to induce lymphopenia than single doses of a maximum of 600 mg cyclophosphamide. In our trial we observed lymphocyte but not neutrophil counts below 200/mm^3 ^in all patients. The question, however, on how lymphopenic patients need to be in order to augment the immune response to an autologous vaccine remains to be answered.

To our knowledge this is the first report to combine lymphopenia, reconstitution and vaccination in the adjuvant clinical setting in lung cancer patients. Powell and coworkers [24] have performed a highly sophisticated trial in melanoma patients where non-myeloablative chemotherapy was combined with an infusion of in vitro-stimulated, gp100 peptide-reactive, autologous PBMC, high-dose IL-2 therapy and vaccination with gp100:209–217(210 M) peptide. No objective clinical responses where observed despite persistence of tumor-specific CD8^+ ^T cells. The authors hypothesize that ineffective cells had been generated by their approach. Jaffee and coworkers [25] combined an allogeneic GM-CSF-secreting vaccine with adjuvant chemoradiation in their phase I trial for patients with surgically resected adenocarcinoma of the pancreas. In this work, no detailed data is presented if these patients were made lympopenic through adjuvant chemoradiation. Nonetheless, this trial may represent an example of vaccination of cancer patients during a phase of lymphoid recovery. Three patients treated at the highest vaccine dose level (≥ 10 × 10^7 ^vaccine cells) seemed to have an increased disease-free survival time, remaining disease-free at least 25 months after diagnosis. Recently, Appay and coworkers published their results on six patients with advanced melanoma treated with lymphodepleting chemotherapy with busulfan and fludarabine, followed by reinfusion of Melan-A specific CD8^+ ^T cell containing peripheral blood mononuclear cells and Melan-A peptide vaccination [26]. One patient presented a partial response on the clinical level. The approach demonstrated good feasibility and low toxicity. However, expansion of Melan-A specific CD8^+ ^T cells in the peripheral blood was mostly inconsistent and the proportion of circulating CD4^+ ^regulatory T cells remained mostly unchanged. Although similar in the underlying approach, several differences such as the treatment of advanced cancer patients, the use of a different lymphodepleting chemotherapy regimen and the application of a peptide vaccine in combination with incomplete Freund's adjuvant instead of GM-CSF will make a direct comparison to our study difficult.

It remains to be seen if our approach using an autologous vaccine in lymphopenic lung cancer patients is able to induce tumor-specific T cells that, in turn, have the capability to find and eliminate potentially remaining tumor cells following surgical resection of the primary tumor. In any case, as illustrated in Figure 5, it is feasible to mimic the preclinical setting described above, in lung cancer patients receiving preparative chemotherapy to induce lymphopenia.

### GM-CSF as vaccine adjuvant

Our data clearly demonstrate that GM-CSF administered as a continuous infusion at the vaccine site via minipump is safe as an adjuvant in this setting. Other groups have used autologous tumor cells (malignant mesothelioma) in combination with repeated subcutaneous injections of recombinant GM-CSF (80 μg) failing to induce major tumor regression despite inducing tumor-specific immunity in 32% of patients [27]. Vaccination with irradiated NSCLC cells genetically modified to secrete GM-CSF has demonstrated safety and significant clinical efficacy [2,3]. However, limitations to this approach might be regulatory hurdles and immune responses directed against the vectors used for transduction of the tumor cells. Furthermore, a threshold effect for vaccine GM-CSF production on survival was noted in these trials, with longer survival seen in patients receiving vaccines secreting >40 ng GM-CSF/10^6 ^cells/24 h [3]. However, attempts to have consistently high GM-CSF secretion at the vaccine site (>2000 ng/10^6 ^cells/24 h) induced by a GM-CSF-producing bystander cell line mixed to the autologous NSCLC cells failed to show association between either tumor cell dose or GM-CSF dose and survival [28]. The authors concluded that adenoviral GM-CSF vectors may provide an adjuvant effect owing to the presence of residual adenoviral proteins in the vaccine that are not present with the bystander approach. While the reason for the failure remains unknown, the significant increase in GM-CSF secretion by the "bystander" GVAX (25 fold higher than the autologous vaccine) may have also had a negative effect. In this context, Serafini and coworkers have reported, that tumor vaccines that secrete high levels of GM-CSF induce myeloid suppressor cells that, in turn, inhibit anti-tumor immunity [29].

With 50 μg/24 h GM-CSF infused at the vaccine site for 6 full days following each of the 5 vaccinations in our trial, we chose a moderate dose of GM-CSF. This was sufficient to induce vaccine site reactions in all patients with the continuous infusion via the osmotic minipump being a safe and feasible means for GM-CSF delivery at a constant dose level. Currently, systemic GM-CSF concentrations over time are being determined in our trial with results pending.

### Clinical course, tumor-related immunity and toxicities

In this paper we establish that induction of lymphopenia followed by reconstitution with autologous PBMC and vaccination with autologous NSCLC cells is both safe and feasible in the adjuvant setting. Immune responses to vaccination were measured by vaccine and DTH skin reactions. Positive vaccine site reactions were observed in 3/3 patients. This is in good accordance with other trials using autologous NSCLC cells as a vaccine, reporting 81% of patients developing vaccine site induration, increasing to 90% after repeated vaccinations [2,3]. As observed in these trials, we saw the most intense vaccine site reaction in patient 2 receiving the highest tumor cell dose. Histopathological assessment of punch biopsy specimens taken at the local vaccine site reaction 48 h following the third vaccine revealed a dense infiltrate consisting predominantly of macrophages, eosinophils, neutrophils and lymphocytes. In contrast to unrelated skin sites, CD1a-positive dendritic cells were present at the vaccine site, most of the infiltrating lymphocytes in the patient with the strongest vaccine site reaction were CD4-positive cells. Further investigation using anti-CD25 and anti-FoxP3 (a marker for regulatory T cells) staining is currently underway to characterize these infiltrating cells.

Positive DTH skin testing was observed in 1/3 patients (again patient 2 with the highest dose of tumor cells) in our trial. This mirrors results from other studies using autologous NSCLC cells as a vaccine approach where the frequencies of positive DTH skin tests clearly correlated with the administered vaccine dose [2,3]. In the study published by Nemunaitis and coworkers [3], DTH testing was positive in 9% of patients at baseline, after 4 vaccinations 34% of patients tested positive. However, no positive DTH reactions were detected at vaccine doses with fewer than 10 × 10^6 ^cells.

The most common vaccine-related adverse events in our study were local vaccine site reactions, followed by constitutional symptoms such as fatigue and mild fever. All injection site reactions consisted of local, self-limiting erythema, induration and pruritus. Overall no grade 3 or 4 adverse events were observed. Patient 2 reported persistent myalgia and arthralgia which was associated with a transient elevation of rheumatoid factor and anti-nuclear antibodies, potentially indicating an autoimmune phenomenon. The same patient also experienced recall responses at the site of previous injections and the baseline DTH injection site following the third vaccination. Most likely, the autologous vaccines manufactured for this trial also contained cells, such as fibroblasts and pneumocytes, and may therefore be able to induce clinically detectable autoimmune symptoms. However, no severe autoimmune disease as observed in other vaccine trials with melanoma patients [8], was observed here. To date, all patients on the trial remain without relapse with the longest follow-up period being 17 months for the first enrolled patient.

## Conclusion

Non-small cell lung cancer is not considered an immune-sensitive malignancy. However, there is increasing evidence that NSCLC can evoke specific humoral and cellular antitumor immune responses. Manipulating the host by preparative chemotherapy and reconstitution with autologous PBMC may augment the vaccine-induced tumor-specific immune response seen in some patients. Here, we report that induction of lymphopenia followed by PBMC reinfusion, combined with an autologous whole tumor cell vaccine and the continuous infusion of GM-CSF at the vaccine site, is a safe and feasible approach in resectable lung cancer patients. Evidence of anti-tumor immunity induced by this approach is present with positive vaccine-site reactions in all patients. However, more patients need to be treated within this protocol in order to be able to assess and compare clinical outcome.

## Competing interests

The author(s) declare that they have no competing interests.

## Authors' contributions

DR and BAF designed the trial protocol, DR coordinated the study and drafted the manuscript. NKE, HW and HP were involved in tumor processing and vaccine preparation. MS supervised chemotherapeutic pretreatment. BW and SG were responsible for leukaphereses harvests, storage and flowcytometric assessment prior to reinfusion (according to GMP). DJS, BAF, KWJ and RAH participated in design and coordination of the study. All authors read and approved the final manuscript.
